# Selections and Abstracts

**Published:** 1898-10

**Authors:** 


### SELECTIONS AND ABSTRACTS.

### WHAT A TRAINED NURSE NEEDS BESIDES A TECHNICAL TRAINING.*

No one can be considered a trained nurse to-day unless she has had the benefit of lectures and lessons, of observation and experience, under the direction of competent teachers. In other words, she must receive a technical training for the work of her chosen calling.

Is there nothing else needed?

Is that technical training enough for her highest efficiency?

Let me suggest some of the other qualifications which every good nurse should have.

*First, the nurse should have a kindly spirit.*

The very vocation of the nurse implies the possession of a kindly spirit.

It is a kindly calling. It must have the response of a kindly soul. She cannot nurse sick people as a woman may keep a set of books in an office, or may sell goods behind the counter of a store. The vocation of the nurse implies the possession of a heart that responds to the cry for help. When a nurse thinks of her vocation as simply a way in which she can earn so many dollars per week, she is astray, just as the clergyman is astray who thinks only of the salary he gets for helping to save men's souls. Sickness not only brings pain and weariness to the patient. It brings usually great trouble to the family. In many a family they do not know what to do, or are unable to do it. The sending out for a nurse is really a cry for help, and it should be regarded as a call to make things better in the afflicted home by bringing in the sunshine of kindliness.

The nurse goes as a womanly woman, to do a greatly needed work for a family in their time of trouble. She is better able to

*Part of an address to the Graduating Class of the Newton Training School for Nurses, June 21, 1898, by Rev. G. W. Shinn, D.D.

do this kindness because she has been trained. She fails if she does not make that home the better for her coming into it. She is to be a gracious presence in that home and is to finish her work in such a way that she will carry with her the good-will of the patient and of all those interested in him. She may make lasting friends of all those to whom she ministers.

*Then, again, the nurse should have tact.*

A good nurse will cultivate that graceful tact which enables her to adapt herself to the varied circumstances into which her calling may bring her. She is to be in homes very differently arranged. They will not all be alike, nor will all be model homes. She will discover in some things which she cannot approve, and in others there will be many a vexatious incident. If she is without tact she will soon come into collision with some one, perhaps every one, and will shortly find herself in the way of all. It requires a great deal of tact to live even for a few weeks in some houses comfortably with strange people. Even when the nurse has the best of intentions and applies herself to her duties, she will be misunderstood and may have things said and done to her of a most provoking character. There is great need of patience and of a proper restraint. There is never anything lost by avoiding all appearance of irritation. She must be quiet and peaceable, and learn to keep silence, and will, in most cases, win her way into favor.

It is a hard thing to describe this quality of adaptability which I have called tact. Some people never acquire it, and some others gain it only after long experience in very small degrees. It is surely worth having, and hence worthy the study and effort of every nurse who would succeed.

*A good nurse must have good sense.*

The good nurse will never lose sight of the fact that there is a singular connection between the mind and the body in disease. Ordinary people are for the time thrown off their balance.

The patient is sick in body. He is also sick in mind. He is not himself as he was last week, not himself as he will be a month hence. He may be most unreasonable now. He will be extremely irritable after awhile. His usual good sense has deserted him.

Do not let your good sense leave you. He will add to your cares by his exhibition of ugly traits which in health he may manage to keep down or out of sight. Sickness often brings a strange infirmity of will, so that the adult has sometimes to be treated like a child; coaxed, implored, expostulated with, encouraged. It would be hard indeed to specify the vagaries which the ordinary sick patient may manifest. The nurse must not be surprised at anything. She will find some of her patients amiable, patient and docile. She will find others just the reverse. And the same patient may be one thing to-day and another to-morrow. A great deal will depend upon her own spirit. She will have to bear up the desponding, and she must brighten the hours of depression. She must bring the sunshine of an amiable manner into the sick-room, and by her strong and hopeful spirit help weaker wills to keep out of the depths.

*The good nurse is loyal.*

The nurse is to regard herself as in some sense the confidential friend of the family, and she carefully guards the family secrets as they become known to her. She may find the skeleton in the closet, but she shuts the door.

These three professions, the law, the ministry and medicine, take special care to guard professional secrets. A lawyer would be disgraced if he revealed the private affairs of his client. The physician has not the right sense of honor who will not guard his patient from too curious observation. The clergyman becomes the trusted custodian of family matters, and is a mean fellow if he does not keep them to himself.

The trained nurse must keep to herself what she hears and sees, if there is anything that comes to her which might be repeated to the disadvantage of the family. Her lips must be sealed as to family matters. She is to see as not seeing, and to hear as not hearing.

It is one element of success in a nurse when she gains the reputation of being discreet. She must be discreet. She cannot fail to learn something in a few weeks' stay in a house that may make trouble if she report it. But she is not to report it. She must lock up in her breast the knowledge that has come to her.

*The trained nurse ought to be a decidedly religious woman.*

Her work is so benevolent, and makes such drains upon her own nature, that I cannot see how one can go on year by year doing the duties of the nurse's calling unless she is actuated by religious motives.

Do not mistake me. I am not saying anything about sectarianism. I am not urging any one to be an adherent of this form of faith or of that, but the ideal nurse will carry Christian principles into her calling, and will make her ministry of the sick a copy of that blessed life which He lived who went about doing good.

A nurse has opportunities for doing good. Her daily conduct, her daily spirit, her daily duties, her daily aims, all may be penetrated by that devotion to the Master Christ which will change all commonplace things into holy service. She can serve the Lord by serving those who are ill. She can do good to bodies and souls in His name. Her opportunities for usefulness are almost unbounded. She is for the time the companion of those who in their feebleness rely upon her. She may win their friendship. She may say the right word at the right time. She may inspire fresh courage and fan the feeble hope into a flame. She may indeed cheer the departing soul as it prepares to wing its flight unto regions unseen, and bid it go forth without fear, because the Son of God opened the gates of life to all who trust in him.

Ah, the religiousness of the vocation of the nurse! Now and then something reveals it to us as never before.

A friend went to a hospital for surgical treatment a year or two ago. In a letter written after his recovery he described his experiences. He says that as he walked up and down the corridors and had glimpses into the wards where lay the sick and the maimed, and saw the tender ministrations bestowed upon them, a sense of the hallowed presence of the Good Physician began to come upon him.

If the gracious Lord were here as once he was in Galilee and in Judea, he would surely be drawn by his tender sympathy for all sufferers to visit the sick and the maimed.

And later on the patient said to himself: "Yes, Lord, the hands of thy servants in this house are doing the same work which thine own blessed hands did when they were laid upon the sick."

And so it came to him that the Lord was present, not visibly present as once he was in homes of sickness and sorrow, but present in the persons and in the ministrations of his servants.

Is not the vocation of a nurse deeply religious, if thus she may be a representative of the Divine Master in tenderness and sympathy?

One day when Benjamin West was painting his great picture of *Christ Rejected*, a friend happened to call and see him. The artist, after the friend had been there a little time, said: "You can do me a good turn to-day. I have reached that point in my picture where I must sketch in the hands. I shall be greatly pleased if you will let me bind your hands together with this cord, and then if you will stand there for some minutes I can finish that part of my picture."

The friend readily consented. His hands were tied together. He stood in the place designated.

For a while the two talked together, but the artist became absorbed in his work and the conversation stopped. The friend, too, stood there in silence. He had no remark to make, no question to ask, for had not the great thought come to him that he was for the time representing Christ?

O you who are privileged to enter this blessed calling, the days are coming when the sick will raise their eyes gratefully as you come near them, because you are to them the representatives of the merciful Christ.

---

### SHOULD THE GENERAL PRACTITIONER RECEIVE A FEE FOR REFERRING CASES TO THE SPECIALIST?

It may seem to a certain few that such a subject should be left out of print. I do not consider it a delicate question, nor one that is best discussed *sub rosa*. I believe that in a certain number of cases the physician should receive a fee for referring cases to the specialist.

The general practitioner is called upon to render every kind of medical and surgical service. If he sees fit to send certain cases to a specialist, he displays a magnanimity unparalleled in other fields of labor. We will admit that in many instances his training and equipment are inadequate in some of the departments now presided over by specialists. The advancement of medicine and surgery has made it difficult for any one man to keep abreast of the entire field. He frequently recognizes that his knowledge in some one department is deficient. He is honest and tells the patient so. The patient is sent to a specialist. There are many instances where the practitioner has found it advisable to administer to his patient for several days before it is possible to refer him to a specialist. Again, in his anxiety for the welfare of his patient he may make several visits in the endeavor to cause the latter to consult the specialist, or may lose valuable time in going with him to the specialist. In any event, he expends a certain amount of his time for which the patient will not compensate him. The patient and his family have long looked upon him as their friend and medical adviser. They never think of paying him for aught but actual professional services. They would not consider such services professional, and he would gain their displeasure if he were to present his bill for the same. In case he has treated the patient for a few day he would better erase any and all such charges for professional services from his books, because the patient considers that he has received no benefit from his treatment; that his physician did not understand his case or he would not have sent him to some one else. Therefore, if this bill is presented no attention is paid to it, and if payment is pressed, the next time a physician is needed some one else is called in. Now, who is to compensate this physician? There is but one answer—the specialist. If the specialist does not pay him for the time he has expended no one else does. The specialist can easily learn from the patient the extent of the services rendered by his physician. The latter should then be compensated accordingly.

There are many times when the family physician is asked to recommend a specialist. He is not called upon for services, nor does he find it necessary to see that the patient goes where directed. He is not inconvenienced nor does he spend any of his time upon the case. Here it is not incumbent upon the specialist to pay the practitioner for his kindness.

Many an obscure practitioner gains standing and even prominence through the specialist. Specialists to whom he refers cases make it a point to speak well of him whenever the opportunity is afforded. This is the only honorable way in which the specialist can return many favors and acts of kindness shown him by the general practitioner.

We all make mistakes, even the general practitioner. What he has done for a given case may not meet with our approval. A hasty word or a sign of disapproval may ruin the reputation of this physician in the eyes of the patient. Hence many cases are not referred because of some such former experience. Again, the time expended on a case has been considerable, to say nothing of the mental worry. To refer the case may mean that all this goes for nothing. The man to whom the case is referred forgets to even say thanks. A few such experiences tend to sour the general practitioner against specialists in general.

The object of this paper is to draw a closer bond of union between the general practitioner and the specialist; to call the attention of the specialist to his duty whenever it is plain.

I do not recommend that physicians be paid a stipulated percentage of all fees received from cases referred by them. I should consider this a great wrong. What I do advocate is that when a physician has expended time and energy in getting a patient to come to us, or has prescribed for the patient without compensation, it should be our duty to pay the physician a reasonable fee.—*Melville Black, M.D., in Colorado Med. Jour.*

---

### EARLY DIAGNOSIS OF TUBERCULOSIS OF THE LUNGS.

Probably no one will question the value of the earliest possible diagnosis of tubercle in the lung, for it is then that help can most easily be given, and it is then that change of environment gives the best hope for the patient. There is little question that the diagnosis of tuberculosis of the lungs is often made by the patient's family before it is recognized by the attending physician. Recognition of the disease in this stage is no credit but shows a most careless observer. To get the best results from treatment it is essential to recognize the pretubercular stage and to treat the patient before the physician can clearly demonstrate, even to his own satisfaction, the undoubted presence of the disease. This is the stage when the tubercle bacillus cannot be shown in the sputum; in fact, the patient has many general symptoms, but few that call attention to the lung. It is this stage that is so often treated for simple anemia, for general debility, for indigestion, and in women for amenorrhea. Even a careful physical exploration, which would put the physician on his guard, is rarely made at this time, as the symptoms do not warrant it, unless the routine practice of examining all patients carefully is adhered to. Yet a physical examination will reveal much; percussion shows little beyond a possible tenderness at some point where the patient has had pain previously. It is believed that this is a little tubercle forming on one of the pleural surfaces, and that the pain and tenderness are the result of a dry pleurisy. Infection does not come from inhalation always, but from infection of the oral cavity, from teeth, breaks in the mucous membranes of tongue, etc., as there is a direct lymphatic connection between the oral cavity and the stomach of the pleura. Auscultation shows possibly a slightly roughened sound, the very faintest of friction sounds heard only after a full inspiration when the patient unconsciously expires a little, though told to hold his breath. This sound heard only at one point is important. Again, a hesitating inspiration or expiration on one side should always suggest the possibility of beginning serious lung trouble. A temperature above normal in the evening, no matter how slight, which is constant, is an important sign of early tuberculosis, provided the morning temperature is normal. A sub-normal evening temperature, while less frequent, is also suggestive of trouble. A persistent cough, which, on most careful examination of the throat, is found due to trouble in the throat, no matter how slight it may be, should always demand a thorough examination of the chest for possible tuberculosis. Of course early examination of the sputum should be made for the tubercle bacillus as soon as there is any expectoration. In a word, where patients complain of getting tired easily, of slowly running down and losing flesh, make a careful physical examination of the chest, even if there is no cough present. It may be the pretubercular stage, and diagnosis in this stage is most important for the patient.—*Charleston Medical Journal.*

---

### CHRONIC ULCERATION (CORNEAL).

Mike Kegan, Stamford; Irish; first seen June 8, 1898. Had been under treatment for three weeks in a New York Hospital, but the disease had continued slowly progressing. When first seen, the ulceration extended, on the right side of the left eye, from almost the center of the corner to and slightly over the sclerotic. Surrounding tissues were tremendously inflamed, and the conjunctiva were ecchymosed. I ordered as the first application, a wet Thiersch pack, to be changed every two hours. After forty-eight hours the inflammation of conjunctiva had almost entirely subsided, and treatment of the ulcer was begun. Patient was put in a horizontal position, and one part hydrozone in two of sterilized water was applied to the ulcer; after which, bovinine pure was dropped on. Nurse was ordered to drop bovinine in the eye every two hours, and every night and morning cleanse the ulcer with hydrozone and water as before. By June 14th, patient was relieved of all pain, which until that time had been extreme. The edges of the ulcer were healing, pushing forth healthy points into the center. June 29th, the ulcer had healed for half of its circumference, and on the corneal side principally. All inflammatory action in the surrounding tissues had subsided, and the ulcer had assumed a thoroughly healthy appearance. Bovinine was continuously applied as before until July 6th, at which time the ulcer had healed with the exception of a small margin between cornea and sclerotic, but this was in a healthy condition and healing steadily. Bovinine was now applied only once in three hours, until July 14th, when the cure was complete and case discharged.—*Sound View Hospital Report.*

---

### VELANDER'S PILLOW-SLIP METHOD OF MERCURIAL INUNCTION.

Dr. R. Hogner describes this new procedure of the Stockholm professor, Dr. E. Velander, for the application of mercurial ointment. He considers it to be practical, effective, convenient, and clean. Velander was convinced, some years ago, that the mercury in an ointment is absorbed after being vaporized. He has, therefore, given up spreading the ointment upon the skin, and spreads it, instead, upon the inside of a small pillow-case, which is hung, upon succeeding days, alternately on the back and on the chest. It is kept in position by ribbons over the shoulders and around the waist. The pillow-case is made of cheap cotton cloth, and one of the external faces is smeared with ointment. It is then inverted so as to bring the ointment on its inner surface, and is placed in position so that the layer of cloth upon which the ointment is spread is in direct contact with the body, while its unsmeared side protects the clothing. Every night fresh ointment is spread over the old without removing this, and at the end of ten days the pillow-case is thrown away and a new one is prepared. Baths should be taken twice a week, and the skin of the chest and back washed every night. The quantity of ointment used daily is six grammes (90 grains). The effect of the treatment is very rapid, and more mercury can in this manner be absorbed with fewer unpleasant effects, such as stomatitis, etc., than when it is otherwise administered.—*Jour. American Med. Assoc.*

---

### IODINE IN OBSTINATE VOMITING.

According to the *Medical News* for July 16th, Steffen recommends the following prescription :

| | | |
|---|---|---|
| ℞ | Tincture of iodine . . . . . . . . . . . . . . . . | 10 drops. |
| | Distilled water . . . . . . . . . . . . . . . . . . . . . . | 4 ounces. |
| M. | | |

One tablespoonful to be taken in half a glassful of sweetened water between meals.

---

### TREATMENT OF IVY POISONING.

The *Canada Lancet* for May recommends the following :

Keep the affected parts well wetted with freshly-made lime-water. Take a teaspoonful four times daily of—

| | | |
|---|---|---|
| ℞ | Fluid extract of couch-grass . . . . . . . . . . . . . . | 4 drachms. |
| | Sweet spirit of niter . . . . . . . . . . . . . . . . . . . | 1 ounce. |
| | Syrup of lemon . . . . . . . . . . . . . . . . . . . . . | 1 " |

### A PAINLESS BLISTER.

The *Journal des praticiens* for June 25th gives the following:

| | | |
|---|---|---|
| ℞ Menthol, Chloral hydrate, | of each | 15 grains. |
| Cacao butter | | 30 " |
| Spermaceti | | 30 " |

Mix to a paste, which may be spread on linen or diachylon plaster. It acts like the cantharides plaster.

### THE RELIEF OF FEVER IN THE TUBERCULOUS.

De Renzi (*Clinica moderna,* June 29th) advises the use of thymol, which has a certain and rapid antipyretic effect without deranging the digestion, but rather improving the condition of the stomach. It is given in doses of four catchets daily, each containing three grains and three-quarters. The dose is augmented until apyrexia is attained. Between ninety and a hundred and five grains suffice to subdue the fever.

### OBEDIENCE.

A gentleman who was suffering severely from business worries and over-anxiety generally, was attended by a well-known physician, whose chief prescription after every visit was a most solemn exhortation to take things easy and not worry about anything.

Ultimately the physician's bill came in, and proved to be of even more than generous proportions. Straightway down sat the patient and wrote precisely as follows:

"Dear Dr. B——: Your account of the 1st inst. duly to hand, and I feel sure that you will be delighted to learn that I am taking things easy and not worrying about anything. Very sincerely yours."—*Doctors' Factotum.*

### FOUR CHLORIDES—GOOD ALTERATIVE.

| | |
|---|---|
| ℞ Arsenici chloridi | gr. j |
| Ammon. chloridi | ʒ ij |
| Tr. ferri chloridi | ʒ iv |
| Hydrargyri chloridi | gr. iss |
| Syrupi | ℥ iij |
| Aquæ ad | ℥ vj |

M. Sig. Teaspoonful three times daily.

